# Evolution, Diversity, and Conservation of Herpetofauna

**DOI:** 10.3390/ani14132004

**Published:** 2024-07-07

**Authors:** Wei Zhu, Bin Wang, Jianping Jiang

**Affiliations:** Chengdu Institute of Biology, Chinese Academy of Sciences, Chengdu 610041, China; zhuwei@cib.ac.cn

Amphibians and reptiles play a critical role in the evolution of Tetrapoda, showcasing significant diversity in terms of their genetics, species, morphology, life history traits, and evolutionary functions [1,2]. These vibrant animals are adapted to a wide range of environments, including extreme habitats such as karst caves and the Qinghai–Tibet Plateau [3,4]. Their adaptability makes them important models for studying the genetic mechanisms underlying speciation [5], environmental adaptation [6,7], and the evolution of crucial functional traits and phenotypes (e.g., air breathing; the amniotic sac) [8], and organ regeneration [9,10].

As essential components of numerous ecosystems, amphibians and reptiles are particularly sensitive to environmental changes, such as warming, pollution, and habitat degradation, due to their limited migration capacity [11,12]. In recent decades, wild populations of amphibians and reptiles have declined in number drastically, with a large proportion of species now considered threatened with extinction [13]. Despite this, the species diversity of amphibians and reptiles remains underestimated, with dozens of new species being discovered each year [1]. Given the acceleration of global changes in climate and environments, there is an urgent need to enrich our understanding of the diversity and evolutionary adaptation of these animals, in addition to how they respond to environmental changes [14,15]. Therefore, it is crucial to develop practical and integrative approaches to guide the conservation of wild amphibians and reptiles [16].

This Special Issue comprehensively addresses amphibian and reptile conservation biology, covering a wide range of topics, including methods for assessing species diversity [17,18,19], genetic diversity, and evolutionary mechanisms [18,20,21,22,23]. It also explores the analysis of environmental adaptation and functional traits [24,25], the impacts of environmental change on these species [26,27], and strategies for their conservation [18,21].

In terms of diversity surveys, eDNA-dependent methods have been successfully applied to detect the presence of amphibian species [19]. This powerful technique has emerged as a critical tool for both qualitative and quantitative surveys of vertebrate populations, progressively establishing itself as a cornerstone method for monitoring the dynamics of vertebrate biodiversity [28]. Notably, three new reptile species are reported in this Special Issue, marking a significant advancement in surveys of amphibian diversity [17,18]. Looking toward the future of this discipline, our goal is to reveal the existence of more species in the future. Genetic diversity and evolution constitute the predominant research theme in this Special Issue, which includes studies on mitogenome diversity and evolution, sex chromosome genotyping, and correlative analysis between genetic diversity and fitness [18,20,21,22,23]. These studies provide mechanistic insights into the biodiversity of herpetofauna. Environmental selection plays a pivotal role in driving genetic variation and diversification in animals. Understanding how amphibians and reptiles adapt to their environments is essential for comprehending the formation and dynamics of genetic diversity. Furthermore, this knowledge provides critical insights into the mechanisms endangering vulnerable species and informs the development of conservation strategies. In the current collection, two studies aim to reveal the physiological or molecular processes associated with environmental adaptation (e.g., semi-arboreal and auditory adaptation) [24,25]. While these findings do not fully convey the underlying mechanisms, they offer crucial clues to what these are. More importantly, these environment-related adaptive traits underscore the potential environmental sensitivity of these species. As climate change continues to intensify, the ability of species to adapt to their environment is increasingly challenged. Investigating the potential impacts of future climate conditions on the environmental adaptation of amphibians and reptiles is a crucial aspect of the field of conservation biology [29]. Studies by Zhang et al. [27] and Xu et al. [26] suggest that climate change is likely to reduce the distribution area of the crocodile lizard and the Alashan pit viper. Finally, Kundu et al. [21] recommend conducting intensive genetic screening on wild and trade/captive star tortoises before translocation, implementing effective enforcement to prohibit wildlife trafficking, and carrying out urgent habitat restoration to conserve this highly threatened species in the wild.

Based on these studies, a structured research framework emerges for amphibian and reptile conservation biology, featuring field surveys, diversity analysis, investigations into environmental adaptation and mechanisms, assessments of the impacts of environmental changes, and the formulation of targeted conservation measures (Figure 1). We hope this framework will enhance the systematicity and depth of research on amphibian and reptile diversity and conservation.

Despite the notable achievements in research on the diversity and conservation of amphibians and reptiles, many questions still require more detailed and thorough answers. With the diversification of sequencing techniques and their decreasing costs, future studies may expand genetic diversity analysis and environmental adaptation to the whole-genome level [30]. Herpetologists might consider initiating an amphibian and reptile genome project with extensive taxonomic and phylogenetic coverage, similar to the efforts made for birds, fish, and mammals. Furthermore, future studies could explore the role of commensal microbiomes in amphibian diversity, environmental adaptation, and conservation. Increasing evidence indicates that the stability and function of host-associated microbiota (e.g., cutaneous and gut microbiota) are crucial to the fitness of animal hosts [31,32,33]. This is also true for amphibians and reptiles [4,34,35]. On the one hand, the susceptibility of host-associated microbiota can mediate the impacts of environmental disturbances on animal hosts [36,37,38]. On the other hand, host-associated microbiota can also help hosts cope with environmental stress [39,40]. For example, skin microbes on frogs prevent morbidity and mortality caused by lethal skin fungi. This insight may represent a target for altering the physiological status of these animals, thereby informing conservation measures for endangered amphibians and reptiles.

## Figures and Tables

**Figure 1:**
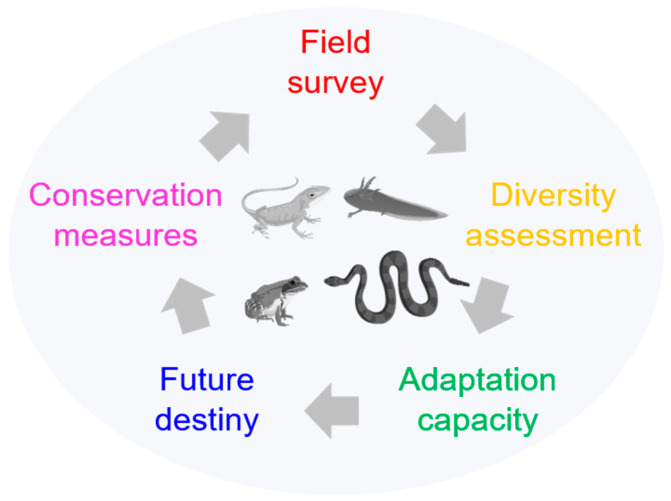
A draft framework for conservation biology research.
